# AGTR1 Is Overexpressed in Neuroendocrine Neoplasms, Regulates Secretion and May Potentially Serve as a Target for Molecular Imaging and Therapy

**DOI:** 10.3390/cancers12113138

**Published:** 2020-10-27

**Authors:** Samantha Exner, Claudia Schuldt, Sachindra Sachindra, Jing Du, Isabelle Heing-Becker, Kai Licha, Bertram Wiedenmann, Carsten Grötzinger

**Affiliations:** 1 Department of Hepatology and Gastroenterology, Charité—Universitätsmedizin Berlin, 13353 Berlin, Germany; samantha.exner@cellphenomics.com (S.E.); schuldt.claudia@gmail.com (C.S.); sachindra.sachindra@charite.de (S.S.); dujing@hmc.edu.cn (J.D.); bertram.wiedenmann@charite.de (B.W.); 2 Department of Gastroenterology, People’s Hospital of Hangzhou Medical College, Hangzhou 310014, China; 3 Institute of Chemistry and Biochemistry, Freie Universität Berlin, 14195 Berlin, Germany; isabelle.heing-becker@fu-berlin.de (I.H.-B.); kai.licha@fu-berlin.de (K.L.); 4 Molecular Cancer Research Center (MKFZ), Charité—Universitätsmedizin Berlin, 13353 Berlin, Germany; 5 Partner site Berlin, German Cancer Consortium (DKTK), 13353 Berlin, Germany; 6 German Cancer Research Center (DKFZ), 69120 Heidelberg, Germany

**Keywords:** neuroendocrine neoplasms, AGTR1, overexpression, signaling, secretion, imaging

## Abstract

**Simple Summary:**

Clinical management of neuroendocrine neoplasms (NEN), especially of those low in target molecules such as somatostatin receptors, may benefit from the discovery of novel targets. This study identified and confirmed angiotensin II (ATII) as a strong activator of signaling in NEN cells and its cognate receptor AGTR1 as overexpressed in human small intestinal NEN. NEN cells with high AGTR1 expression exhibited cellular activation and secretion upon stimulation with ATII. AGTR1 ligand saralasin coupled to a fluorescent dye demonstrated tumor accumulation in an animal model of NEN. This proof of concept establishes AGTR1 as a novel target in NEN, paving the way for its potential use in diagnostic PET imaging and radioligand therapy.

**Abstract:**

This study identified and confirmed angiotensin II (ATII) as a strong activator of signaling in neuroendocrine neoplasm (NEN) cells. Expression analyses of the ATII receptor type 1 (AGTR1) revealed an upregulation of mRNA levels (RT-qPCR) and radioligand binding (autoradiography) in small-intestinal (*n* = 71) NEN tissues compared to controls (*n* = 25). NEN cells with high AGTR1 expression exhibited concentration-dependent calcium mobilization and chromogranin A secretion upon stimulation with ATII, blocked by AGTR1 antagonism and Gαq inhibition. ATII also stimulated serotonin secretion from BON cells. AGTR1 ligand saralasin was coupled to a near-infrared fluorescent (NIRF) dye and tested for its biodistribution in a nude mouse model bearing AGTR1-positive BON and negative QGP-1 xenograft tumors. NIRF imaging showed significantly higher uptake in BON tumors. This proof of concept establishes AGTR1 as a novel target in NEN, paving the way for translational chelator-based probes for diagnostic PET imaging and radioligand therapy.

## 1. Introduction

Neuroendocrine neoplasms (NENs) represent a heterogeneous group of neoplasms with a moderate but steadily increasing annual age-adjusted incidence of approximately 7/100,000 for the USA in 2012 [1]. Most NENs grow rather slowly and may develop into a more aggressive phenotype while being undetected. Somatostatin analogs (SSAs) such as octreotide are used as antisecretory and antiproliferative medication. Apart from direct pharmacological intervention, SSTR overexpression in NENs has also been utilized for targeted molecular imaging and peptide receptor radiotherapy (PRRT) [2,3,4,5]. However, up to 30% of patients do not fully benefit from these approaches. While some tumors lack SSTR expression or downregulate expression upon treatment, others exhibit an inhomogeneous receptor distribution resulting in a residual tumor mass after treatment and subsequent resistance to SSAs [6,7]. Other possible resistance mechanisms include the following: receptor desensitization, downregulation or loss-of-function as well as altered signaling pathways [7,8]. Most patients develop SSA resistance within weeks to months of treatment, with recurrent symptoms and tumor growth [9]. 

To identify alternative target receptors at the cell surface of NENs, this study used high-throughput screening technology to detect G protein-coupled receptor (GPCR) activation in human NEN cells. The screening revealed a clear response towards angiotensin II (ATII). Therefore, this study went on to assess the potential of AGTR1 as a novel target in NEN. Study objectives were to validate AGTR1 mRNA expression and ligand binding in human NEN tissue, to investigate the biological effects of ATII in human cell models, and to evaluate the suitability of AGTR1 as a target for tumor diagnosis in a mouse xenograft model.

## 2. Results

**Figure 1:**
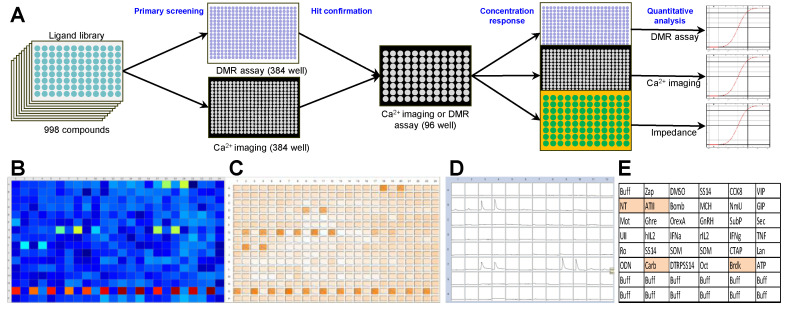

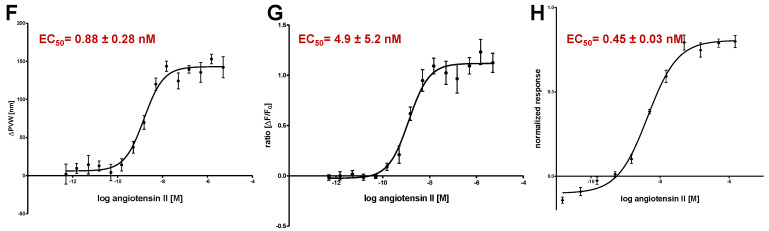
Identification and confirmation of ATII-induced signaling in neuroendocrine neoplasm (NEN) cells. (**A**) Schematic workflow of the screening and hit confirmation approach. A 998-compound library of peptides and small molecules was used in the primary screening on neuroendocrine BON cells, applying both dynamic mass redistribution (DMR) and calcium mobilization assay in the 384-well format, followed by hit confirmation using the calcium assay (96-well format). Finally, DMR, calcium and impedance assays were used to determine quantitative pharmacological data. (**B**) Pseudocolor representation of the screening result from one DMR assay plate. Duplicates are interspaced by one well, penultimate row contains positive and negative controls. (**C**) Representative results from calcium assay, plate layout as in (**B**). (**D**) Plate view of the hit confirmation calcium assay with fluorescence intensity traces for each well. (**E**) Plate layout with ligands used for hit confirmation, plate as in (**D**). (**F**–**H**) Concentration–response curve for angiotensin II in BON cells measured by DMR assay (**F**), calcium mobilization (**G**) and impedance (**H**).

A 998-compound chemical library mainly consisting of peptide receptor ligands (Table S1) was applied in a primary screening in the NEN cell line BON. Both a label-free dynamic mass redistribution (DMR) as well as an intracellular calcium mobilization assay were employed in 384-well format (Figure 1A–C). A total of 89 hits were detected (8.9%), which were assayed again in BON cells in a 96-well format (Figure 1D,E). Thirty primary hits, including structurally related compounds, were confirmed in 96-well format in the DMR assay, and 19 hits in the calcium assay, with 11 compounds confirmed in both assays (Table S2). Angiotensin II (ATII) appeared consistently as a strong activator of intracellular signaling in BON cells. This was subsequently validated in three functional assays: DMR, calcium and impedance. Quantitative analysis showed a half maximal effective concentration (EC_50_) for ATII of 0.88 ± 0.28 nM, 4.9 ± 5.2 nM and 0.45 ± 0.03 nM in DMR, calcium and impedance, respectively (Figure 1F–H). 

**Figure 2:**
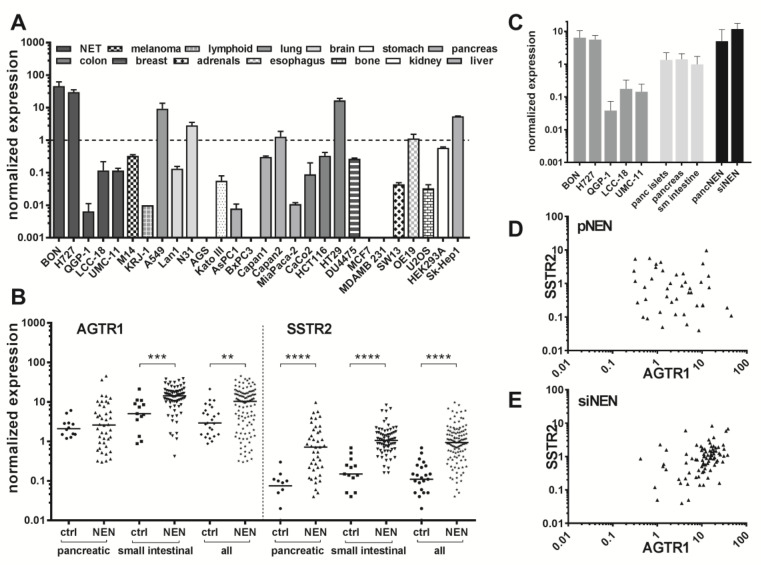
Increased AGTR1 gene expression in pancreatic and small intestinal NEN tissue and two NEN cell lines. (**A**) AGTR1 expression was measured as mRNA levels by RT-qPCR in 28 cell lines of different origin. Values were normalized on UBC, HPRT1 and GAPDH. Bars show mean ± SD (*n* = 2, different passages). (**B**) AGTR1 mRNA levels determined by RT-qPCR in pancreatic (*n* = 42) and small intestinal (*n* = 71) NEN tissues in comparison to pancreatic (*n* = 12) and small intestinal (*n* = 13) control tissues. Values were normalized on ALG9, UBC and HPRT1 and compared to SSTR2 gene expression in the same samples. Bars represent median. Evaluated with unpaired two-tailed Student’s *t*-test, ** *p* < 0.01, *** *p* < 0.001, **** *p* < 0.0001. (**C**) Direct comparison of AGTR1 expression in NEN cell lines (dark grey bars), normal tissues (pancreatic islets, pancreas, small intestine; light grey bars) and NEN tissue (black bars). (**D**,**E**) Scatter plots showing the correlation of AGTR1 with SSTR2 gene expression in pancreatic (pNEN) and small intestinal (siNEN) tumor tissues.

Endogenous expression of the ATII receptor AGTR1 was assessed in five NEN cell lines by RT-qPCR and compared to mRNA levels of 23 other tumor and non-tumor cell lines (Figure 2A). NEN cell lines BON and H727 exhibited the highest mRNA levels of all tested, indicating an AGTR1 overexpression in NEN. The NEN cell line QGP-1 showed an at least 1000-fold lower AGTR1 mRNA expression, while LCC-18 and UMC-11 had intermediate expression levels. Pancreatic (*n* = 42) and small-intestinal (*n* = 71) tumor tissues of NEN patients were analyzed for their AGTR1 mRNA levels. To allow a direct comparison with their organs of origin, normal control tissues (*n* = 25) were also included. Small-intestinal NEN (siNEN) demonstrated significantly (*p* < 0.001) increased AGTR1 transcript levels, with an overall 2.8-fold higher median value in comparison to healthy control tissues, whereas pancreatic tissues showed no significant difference (Figure 2B). Taking the median value of the siNEN samples as a cut-off, 50% of tumor samples yet only 7.7% of controls were above that value. When all NENs were compared to all controls, differences were also significant (3.6-fold higher median, *p* < 0.01). No correlation of AGTR1 mRNA levels with stage, grade or Ki67 value were found (see Figure S1 for correlation analysis with Ki67). To allow a direct comparison, AGTR1 mRNA levels in NEN cell lines, normal controls and NEN tumor tissue were analyzed and plotted. Normal pancreas, pancreatic islets and small intestine showed similar mRNA levels. Higher levels in pNEN and siNEN samples matched well those of NEN cell lines BON and H727, while the other NEN cell lines were found to have lower mRNA levels (Figure 2C). The same clinical samples were also analyzed for expression of the established NEN target SSTR2, which was found to be significantly upregulated in both pancreatic and siNEN tissues, as expected. The ratio of NEN to control median values was higher for SSTR2 (8.5 versus 3.6 for AGTR1). However, AGTR1 was detected at an approximately 10-fold higher expression level than SSTR2. Correlation analysis confirmed a significant positive correlation between AGTR1 and SSTR2 expression for small-intestinal NENs (Pearson *r* = 0.37, *p* < 0.001, *r*^2^ = 0.14), whereas no significant correlation was found for pancreatic samples (*r* = −0.09) (Figure 2D,E).

**Figure 3:**
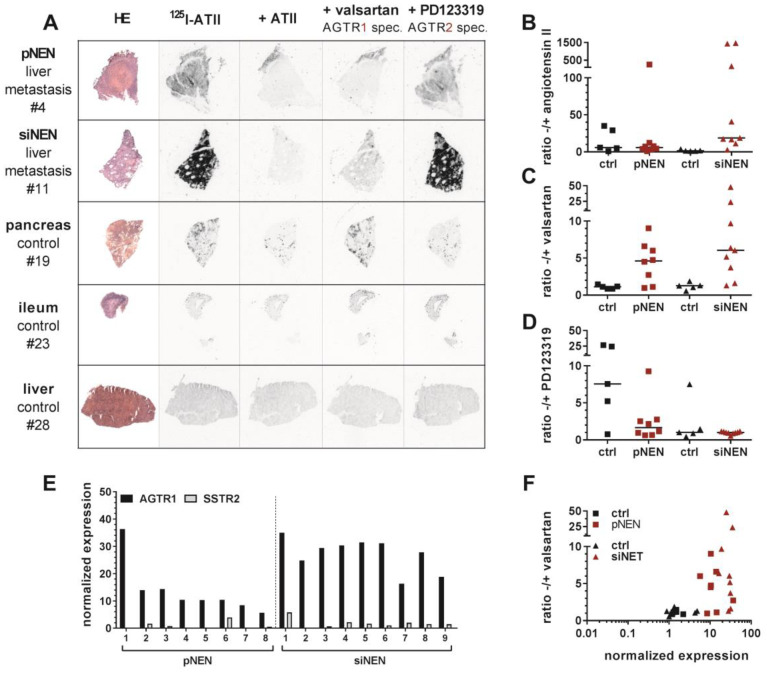
In vitro receptor autoradiography confirms increased AGTR1, not AGTR2 expression in pancreatic and small intestinal NENs. From all samples that were analyzed by RT-qPCR, 10 control and 17 NEN samples were used for autoradiographic evaluation of receptor expression. (**A**) Hematoxylin and eosin (H&E) staining and autoradiograms of representative pancreatic (pNEN) and small intestinal (siNEN) tumors as well as of pancreatic, small intestinal (SI) and liver control tissues. For each tissue, consecutive cryosections were either incubated with iodine-125 labeled angiotensin II alone (^125^I–ATII, total binding) or in the presence of additional 1 µM unlabeled ATII, AGTR1 antagonist valsartan or AGTR2 antagonist PD123319 (non-specific binding). (**B**–**D**) For all autoradiograms, mean signal intensities per area were calculated and are shown as ratios of total to non-specific binding for ATII (**B**), valsartan (**C**) and PD123319 (**D**). (**E**) Scatter plot showing the correlation of AGTR1 gene expression (x-axis, RT-qPCR) with receptor protein expression (y-axis, autoradiography). (**F**) For the analyzed NEN tissue samples, AGTR1 gene expression levels were compared to their SSTR2 expression, as measured with RT-qPCR (see Figure 2).

To verify AGTR1 gene expression employing an alternative methodology, ten normal control samples from pancreas and small intestine as well as eight pancreatic and nine small-intestinal NEN samples were selected for the determination of receptor binding sites by in vitro receptor autoradiography (patient characteristics: Table S3). To this end, the natural receptor ligand ATII was radioactively labeled with iodine-125 (^125^I–ATII) and purified by high-performance liquid chromatography (HPLC) (Figure S2). Consecutive cryosections of each tissue were incubated with the radioligand in the absence or presence of an excess of non-labeled ATII. To distinguish between receptor subtypes, additional tissue sections were incubated with radioligand and AGTR1-specific antagonist valsartan or AGTR2-specific antagonist PD123319 (Figure 3A, Figures S4 and S5). Pancreatic NEN tissues showed a rather weak overall binding of ^125^I–ATII. Nevertheless, in tissues #4 (Figure 3A), #1, #6 and #8 (Figure S4), it could be clearly displaced by ATII and valsartan, but not by PD123319, indicating dominant AGTR1 expression. Small-intestinal NENs, on the other hand, demonstrated a strong binding in more than half of the samples (#9 to #13) (Figure 3A, Figure S4). This proved to be AGTR1-specific binding as it could only be displaced by valsartan. PD123319 was unable to compete with the radioligand. In addition, pancreatic and small-intestinal control tissues were included in the analysis (Figure 3A, Figure S5). Interestingly, a number of pancreatic samples exhibited strong radioligand binding. In comparison to pancreatic NEN samples, signals in normal pancreas were displaced solely by PD123319, indicating a specific expression of AGTR2. Small-intestinal control samples showed no binding at all (#23, #25) or non-specific binding (#24, #26, #27) (Figure S5). 

Digitized autoradiograms were quantified by calculating the ratios of total binding to non-specific binding in the presence of excess ATII, valsartan or PD123319 (Figure 3B–D). The higher a ratio, the higher the expression of the respective receptor subtype. Indeed, valsartan-related ratios were increased up to 5-fold in NEN tissues, especially in small-intestinal NENs, when compared to their respective control tissues. This confirmed a specific AGTR1 expression (Figure 3C). To verify these findings using a different quantitative analysis, tissues were wiped off the slides after radioligand incubation and measured by a gamma counter. This alternative quantification yielded similar results (Figure S3). As depicted in Figure 3F, mRNA levels correlated well with binding levels for most tissues (Pearson *r* = 0.43, *p* ≤ 0.01, *r^2^* = 0.184). While controls showed low mRNA and low binding levels, NENs in general had relatively high mRNA and binding levels. A similar correlation using the alternative quantification confirmed this result (Figure S3B). Furthermore, AGTR1 mRNA levels of the samples analyzed by autoradiography were individually compared to those of SSTR2 mRNA (Figure 3E. All these NEN tissues showed higher AGTR1 than SSTR2 mRNA levels.

**Figure 4:**
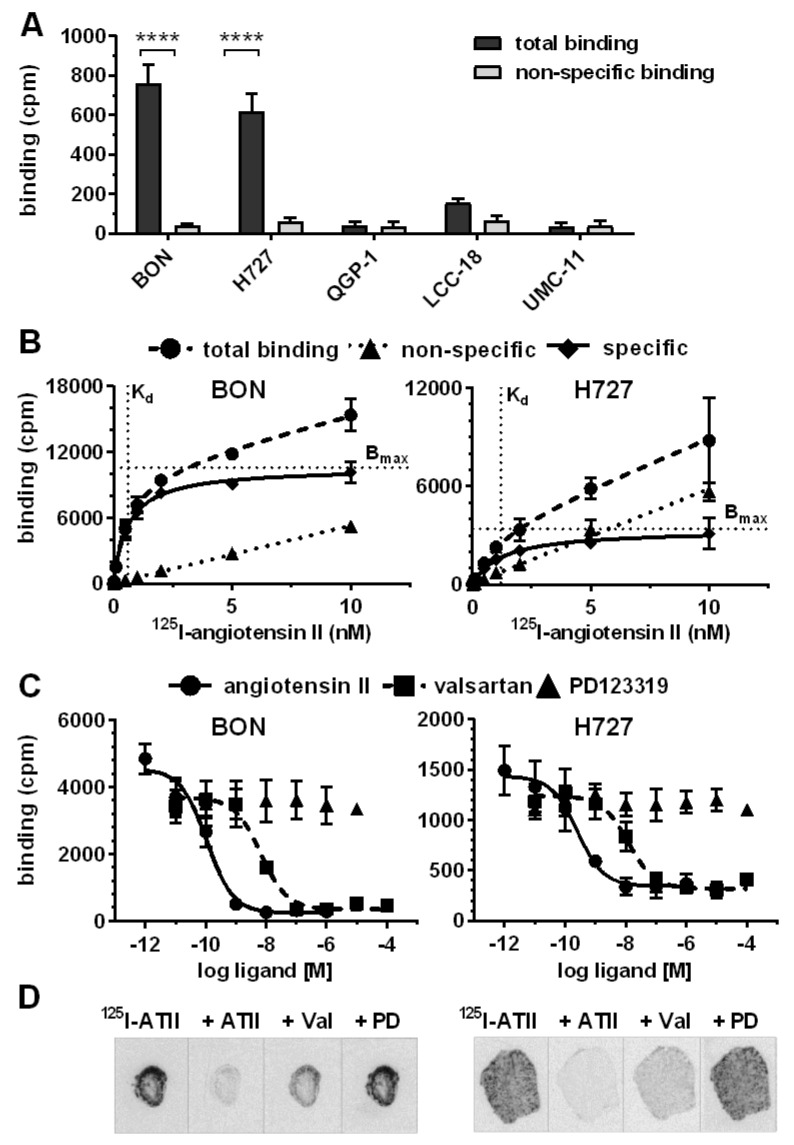
NEN cell lines BON and H727 specifically express AGTR1, not AGTR2. (**A**) NEN cell lines were incubated with ^125^I–ATII alone (total binding) or in the presence of additional 1 µM unlabeled ATII (non-specific binding). Data represent mean ± S.E.M. (*n* = 3). Evaluated with two-way ANOVA, **** *p* < 0.0001. (**B,C**) Saturation (**B**) and competition (**C**) binding assays were performed for BON and H727 cells. Data represent mean ± S.E.M. (*n* = 3). (**B**) Cells were incubated with increasing concentrations of ^125^I–ATII in absence (total binding) or presence of 1 µM unlabeled ATII (non-specific binding). Specific binding was calculated by subtraction of non-specific from total binding. (**C**) Cells were incubated with ^125^I–ATII and increasing concentrations of either unlabeled ATII, valsartan (AGTR1 antagonist) or PD123319 (AGTR2 antagonist). (**D**) Autoradiograms of BON and H727 xenografts showing total (^125^I–ATII) or non-specific binding in the presence of 1 µM ATII, valsartan or PD123319. cpm, counts per minute.

After target validation in patient tissues, receptor-positive cell lines BON and H727 were further evaluated as models for AGTR1 expression and function in NEN. Confirming gene expression data (Figure 2A), both cell lines exhibited significant specific binding of ^125^I–ATII that could be displaced by unlabeled ATII, whereas the other NEN cell lines QGP-1, LCC-18 and UMC-1 demonstrated only background levels of radioligand binding (Figure 4A). Saturation radioligand binding experiments determined K_d_ values of 0.6 nM for BON and 1.2 nM for H727 (Figure 4B). In contrast, the cell lines differed in their receptor density as indicated by the B_max_ value, which was 3-fold higher for BON (~50,000 binding sites/cell) in comparison to H727 cells (~16,000 binding sites/cell). As depicted in Figure 4C, binding of ^125^I–ATII to BON and H727 cells could be displaced in a dose-dependent fashion by ATII and the AGTR1-specific antagonist valsartan, but not by the AGTR2-specific antagonist PD123319. Calculated K_i_ values reflect the expected high affinity of ATII for AGTR1 (BON: 0.1 nM, H727: 0.2 nM). K_i_ values of valsartan were slightly higher, but still in the low nanomolar range (BON: 4.3 nM, H727: 9.4 nM). It was further shown that both cell lines retained AGTR1 expression and radioligand binding capacity during xenotransplantation and tumor growth in nude mice, as analyzed by receptor autoradiography (Figure 4D).

**Figure 5:**
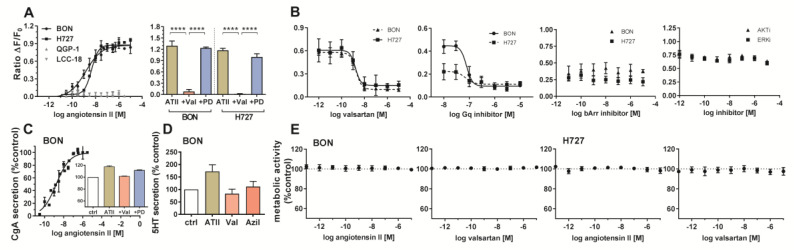
Calcium signaling, chromogranin A secretion and viability of NEN cell lines after stimulation of AGTR1. (**A**) NEN cell lines were preloaded with the calcium indicator fluo-4, and its increasing fluorescence after application of ATII was recorded (left). BON and H727 were additionally pretreated with 1 µM valsartan or PD123318 for 15 min before ATII stimulation (right). Data show mean ± S.E.M. (*n* ≥ 3). Evaluated with two-way ANOVA, **** *p* < 0.0001. (**B**) BON and H727 cells were preloaded with the calcium indicator fluo-4 and preincubated with either valsartan, Gαq blocker UBO-QIC, β-arrestin blocker barbadin or inhibitors for ERK or AKT (BON cells) for 15–30 min. Fluorescence intensity after application of ATII was recorded. Data show mean ± S.E.M. (*n* ≥ 3). (**C**) BON cells were either treated with increasing concentrations of ATII (main figure) or pretreated for 15 min with 10 µM valsartan or PD123318 before application of 100 nM ATII (inset). Chromogranin A (CgA) content of the supernatants was measured after 6 h (left) or 24 h (right), respectively. Data show mean ± S.E.M. (*n* ≥ 2). Values were normalized on untreated controls. (**D**) BON cells were treated with either 1 µM ATII, valsartan or azilsartan. Serotonin (5HT) content of the supernatants was determined after 6 h. Values were normalized on untreated controls. Data show mean ± S.E.M. (*n* = 3). (**E**) BON and H727 cells were incubated with increasing concentrations of angiotensin II or valsartan for 96 h and analyzed for their metabolic activity by addition of AlamarBlue. Data show mean ± S.E.M. (*n* = 4).

Cellular signaling, secretion and cell viability of BON and H727 cells were evaluated upon receptor stimulation or inhibition. Receptor activation was studied by an intracellular calcium mobilization assay. While concentration-dependent response curves could be obtained for BON and H727, demonstrating functionally active receptor in these cells, QGP-1 and LCC-18 showed no calcium mobilization (Figure 5A). Furthermore, valsartan was able to significantly diminish the induced signal, while PD123319 did not influence receptor activation, indicating selective AGTR1, not AGTR2 signaling (Figure 5A). Valsartan inhibited ATII-mediated calcium mobilization in a concentration-dependent manner (Figure 5B). Gα_q_ inhibition completely abolished the signal, whereas β-arrestin blockage did not modulate the effect. Inhibition of ERK or AKT also did not change the calcium response in BON cells (Figure 5B).

BON cell supernatants were tested for their chromogranin A (CgA) content after ATII treatment. Interestingly, CgA secretion in these cells was stimulated in a concentration-dependent manner. CgA secretion was diminished after preincubation with AGTR1 antagonist valsartan (Figure 5C). Moreover, ATII treatment stimulated the release of serotonin (5HT) to the cell supernatant, while application of the receptor blockers valsartan and azilsartan did not modify basal 5HT secretion (Figure 5D). When investigating metabolic activity as a readout for cell growth and viability, no change could be detected after 96 hours of treatment with different concentrations of agonist (ATII) or antagonist (valsartan) (Figure 5E).

**Figure 6:**
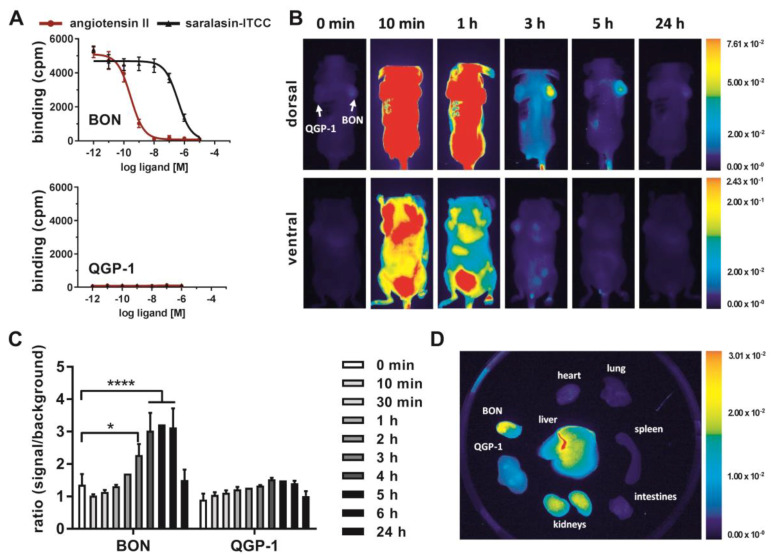
In vivo near-infrared fluorescent (NIRF) imaging with AGTR1-targeting saralasin–ITCC. (**A**) AGTR1-positive BON and AGTR1-negative QGP-1 cells were incubated with ^125^I–ATII and increasing concentrations of unlabeled ATII or saralasin–ITCC. Data show mean ± S.E.M. for BON (*n* = 3–4) or mean ± S.D. for QGP-1 (*n* = 1). (**B**) Biodistribution of 1 nmol i.v. saralasin–ITCC in a mouse model subcutaneously injected with BON (right shoulder) and QGP-1 cells (left shoulder). Images were acquired at the indicated time points before or after injection and are displayed with an equally adjusted gain for either dorsal or ventral signals. (**C**) Signals from in vivo NIRF imaging were quantified by calculating the ratio of signal (tumor) to background (neck) for saralasin–ITCC. Bars represent mean ± S.E.M. of four different animals. Evaluated with matched two-way ANOVA and Bonferroni post hoc test, * *p* < 0.05, **** *p* < 0.0001. (**D**) Ex vivo NIRF imaging of tumors and organs 6 h after injection, exemplary images from one animal. cpm, counts per minute; ITCC, indotricarbocyanine.

Finally, the capacity of AGTR1 to act as a target for in vivo molecular diagnostic imaging was assessed. To this end, the partial AGTR1/2 agonist saralasin was coupled via a 4,7,10-trioxatridecan-succinamic acid (TTDS) linker to the near-infrared fluorescent dye indotricarbocyanine (ITCC). Radioligand binding assays with ^125^I–ATII were conducted to determine the affinity of the fluorescent probe in vitro (Figure 6A). In AGTR1-positive BON cells, the radioligand could be displaced by unlabeled ATII confirming its high affinity (K_i_ = 0.1 nM) as determined before (Figure 4C). The binding curve of saralasin–ITCC was shifted to the right, indicating lower binding affinity at a K_i_ value of 246.5 nM. In contrast, receptor negative QGP-1 cells did not show binding at all. For in vivo imaging, immunodeficient nude mice were subcutaneously inoculated with BON cells on the right and QGP-1 cells on the left shoulder. Near-infrared fluorescent imaging of live animals was performed after sufficient tumor growth, and the ITCC-labeled probe was tested in four different animals. Biodistribution kinetics of saralasin–ITCC of one animal after intravenous application is shown in Figure 6B. Visual evaluation of the images indicated a selective accumulation of saralasin–ITCC in AGTR1-positive BON tumors, which proved to be statistically significant at three, four, five and six hours post-injection (Figure 6C). Tumor-to-background ratios were 2- to 3-fold higher when compared to the image acquired prior to injection. Low non-significant uptake was detected in receptor negative QGP-1 tumors. Ex vivo organ imaging 6 hours after injection confirmed the specificity of saralasin–ITCC for receptor-positive BON tumors as well as an accumulation in kidneys and liver (Figure 6D). Similarly, AGTR1 small molecule antagonist valsartan was labeled with ITCC via a 1,3-diamino propane linker and used for the same type of imaging. Although the affinity of this fluorescent ligand conjugate (IC_50_ = 18.6 nM) was higher than for saralasin–ITCC, the accumulation of valsartan–ITCC in AGTR1-positive NEN xenograft tumors proved to be less pronounced (Figure S6).

## 3. Discussion

In search of alternative targets that may be used for diagnosis and treatment of NENs, this study employed cell-based screening in human NEN cells using a compound library of 998 peptides and small molecules, yielding 38 confirmed hits, including ATII and a number of other GPCR ligands. These results stress the validity of activity-based screening approaches (reverse genetics) to identify new targets in oncology. In a comprehensive analysis of AGTR1 expression in 123 NEN patient and 25 control samples, this study demonstrated an upregulation of AGTR1 in small-intestinal NENs. While absolute expression in cancer tissue may be the most important criterion for judging the usefulness of a theranostic target, comparison with expression in normal tissues can be indicative of potential background signals in the organs of origin (pancreas, small intestine). Correlation analyses revealed a positive correlation between AGTR1 and SSTR2 expression in these samples. However, a subset of patients with low SSTR2 and high AGTR1 expression could benefit from tumor targeting approaches based on AGTR1 ligands. Data for the other SSTR family members have not been obtained. However, as SSTR2 is dominant for targeting in terms of both tumor expression as well as somatostatin analogue affinity, the contribution of other SSTRs to the imaging success would probably be limited. While mRNA expression data provide important insights into differentially regulated transcript levels, the existence of sufficient receptor protein at the cell surface is a prerequisite for molecular targeting. In a previous study, AGTR1 protein expression in 44 pancreatic NENs had been evaluated by immunohistochemistry [10]. This paper observed an expression in 80% of the patients. However, the antibody employed in this paper was demonstrated to be non-specific in two studies [11,12]. Due to the lack of validated AGTR1 antibodies, in the current study, protein expression was indirectly evaluated by receptor autoradiography as an alternative method. Quantification revealed an up to 5-fold higher binding of AGTR1 in particular in small-intestinal NENs. This was found to correlate well with mRNA expression for most samples. 

In a panel of 28 cell lines tested for AGTR1 expression, the two cell lines with the highest AGTR1 mRNA expression were of NEN origin, whereas only one colon and one lung cancer cell line reached similarly high values. This may indicate that AGTR1 overexpression is not a general phenomenon in tumors, but may be of particular relevance to NENs. Specific AGTR1 expression in these cell lines was independently confirmed by radioactive binding assays and autoradiography.

Several studies showed that ATII facilitates cell proliferation in various human cancer cell lines, including breast [13], prostate [14] and pancreatic cells [15]. The proposed proliferative effect, however, could not be confirmed in this study, as metabolic activity was not affected. Likewise, treatment with the AGTR1 antagonist valsartan revealed no inhibition of cell growth. BON and H727 might release ATII, leading to autocrine signaling and enhanced cell proliferation, making externally added treatment negligible. This would be in line with a previous study in which treatment with the ACE inhibitor enalapril, and thereby inhibition of ATII production, resulted in decreased BON cell growth in vitro and in vivo [10]. Similarly, the effect of applied antagonists might be impaired as they have to compete with autocrine ATII binding for the receptors [16]. Furthermore, ATII mediates its proliferative signaling not only by direct stimulation of tumor cells, but also indirectly affects stromal and vascular cells [17], which are not present in most monolayer cell cultures. 

Finally, BON cells harbor a number of untypical mutations (e.g., in TP53, TSC2) [18] that may drive non-responsiveness of proliferation to regulation by AGTR1 agonists and antagonists. Similarly, QGP-1 cells are known for a number of mutations that are not typical for well-differentiated neuroendocrine tumors. This clearly constitutes a limitation of the experimental approach, as does the fact that it is a pancreatic NEN cell line (BON) that is strongly AGTR1-positive, while, overall, pancreatic NEN show no significant overexpression. Unfortunately, an AGTR1-overexpressing siNEN cell line that could be employed in the in vivo imaging experiments was not available. In contrast to other tumor entities like breast cancer or colorectal cancer with hundreds of cell lines, in the field of the heterogeneous neuroendocrine neoplasms, comparatively few cell lines have been developed, and almost none is available in public repositories. These cell lines do not at all recapitulate the landscape of these tumors. The lack of data for lung NEN is another limitation of this study, which was focused on NEN in the gastroenteropancreatic system. The scarcity of cell model systems in the field is currently improving step by step [19], and more novel cell lines may become available with a more typical tumor biology. Primary cell cultures in 2D or 3D as well as patient-derived xenografts may pave the way for more appropriate and authentic NEN in vitro and in vivo models. 

Radiolabeled AGTR1-targeted peptides and small molecules have been primarily utilized for cardiac and cardiovascular imaging to date, to select patients for distinct treatment options and to better predict therapy response [20]. To the best of our knowledge, this is the first study investigating the applicability of AGTR1-based tumor targeting. Biodistribution of the two different ITCC-labeled targeted ligands was evaluated in a xenograft mouse model, revealing a significant accumulation of saralasin–ITCC in receptor-positive BON tumors. The superior performance of saralasin–ITCC was unexpected, as the introduction of the dye molecule ITCC decreased its affinity 250-fold when compared to the published K_i_ value of 1 nM [21]. Although a slow dissociation rate from the receptor is mostly associated with high affinity binding, both parameters do not necessarily have to correlate [22]. For a translation of this targeting principle into a clinically relevant theranostics approach, the affinity of a tracer will have to be improved, e.g., by coupling the chelator to another site in the molecule or by the introduction of another spacer. 

## 4. Materials and Methods 

### 4.1. Reagents

If not indicated otherwise, chemicals were obtained from Sigma Aldrich (St. Louis, MO, USA). G_αq_ inhibitor UBO-QIC was purchased from the Institute of Pharmaceutical Biology, University of Bonn (Bonn, Germany), AKT-Inhibitor AZD-5363, #A9601, from LKT laboratories (St Paul, MN, USA), ERK-Inhibitor SCH772984, #B1682-5, from BioVision (Milpitas, CA, USA), and β-arrestin inhibitor barbadin, #Axon2774, from Axon Medchem (Groningen, The Netherlands). 

### 4.2. Compound Library

The compound library used for screening consisted of the following components: compounds 1–866 were from the G-Protein Coupled Receptor (GPCR) Peptide Ligand Library (Phoenix Pharmaceuticals, Burlingame, CA, USA); compounds 867–956 from the Orphan Peptide Ligand Library (Qventas Labs, Branford, CT, USA), and compounds 957–998 from various sources. All compounds in the library are listed in Table S1.

### 4.3. Tissues

This study was carried out in accordance with the recommendations and protocol approval by the local ethics committee at Charité—Universitätsmedizin Berlin with written informed consent from all subjects, in accordance with the Declaration of Helsinki. Samples were flash-frozen after surgical resection and stored at −80 °C until further use. Of 42 pancreatic NEN samples, 7 (17%) were grade 1, 27 (64%) were grade 2 and 8 (19%) were grade 3. Seventeen (40%) of the pNENs were from metastatic samples; 25 (60%) were from primary tumor samples. The mean Ki67 value for the pNENs was 14.24 (95% CI 7.73–20.75). Of 71 small intestinal NEN samples, 38 (53.5%) were grade 1, 32 (45%) were grade 2, and 1 (1.5%) was grade 3. Twenty-five (35%) of the siNENs were from metastatic samples; 46 (65%) were from primary tumor samples. The mean Ki67 value for the siNENs was 3.92 (95% CI 2.44–5.39). 

### 4.4. Reverse Transcription Quantitative Real-Time PCR (RT-qPCR)

RNA isolation, reverse transcription and quantitative real-time PCR were performed as previously described [23]. Oligonucleotide primer sequences are indicated in Table 1. Plotted values were corrected for different primer efficiencies and normalized on the geometric mean of UBC, HPRT1 and ALG9 or GAPDH using the ΔΔCt method [24]. Reference genes were validated and chosen using the geNorm algorithm [25] by qbase+ software (Biogazelle, Ghent, Belgium).

### 4.5. AGTR1 Ligands and Ligand Conjugates

Angiotensin II as well as saralasin-ITCC (the dye indotricarbocyanine (ITCC) linked via 4,7,10-trioxatridecan-succinamic acid (TTDS)) were obtained from peptides&elephants (Hennigsdorf, Germany). Valsartan and PD123319 were from Tocris (Bristol, UK). Valsartan–ITCC (the dye ITCC linked via 1,3-diamino propane) was synthesized as described in the Supplementary Methods Subsection. 

### 4.6. Other Methods and Data Availability

Further methods are described as part of the Supplementary Material. Numerical data for all applicable experiments (xlsx file) have been deposited in an open data repository for public access: http://doi.org/10.5281/zenodo.4009499. 

## 5. Conclusions

The proof of principle for AGTR1-based tumor imaging in this study may pave the way for a translation into diagnostic approaches like PET imaging and AGTR1-directed peptide receptor radioligand therapy (PRRT) for NENs employing chelator-based AGTR1 radiotracers. 

## Figures and Tables

**Table 1:** Primer sequences used for RT-qPCR.

Gene	Primer forward Sequence	Primer Reverse Sequence	Amplicon Length (bp)
AGTR1	TTTTCGTGCCGGTTTTCAGC	TGCAACTTGACGACTACTGC	100
SSTR2	CCCCTCACCATCATCTGTCT	AGGTGAGGACCACCACAAAG	247
UBC	ATTTGGGTCGCAGTTCTTG	TGCCTTGACATTCTCGATGGT	133
ALG9	GTCTTCTGGCTTTTGTGAGCTG	TCACGTGCAACCCAAACTTC	78
HPRT1	TGACACTGGCAAAACAATGCA	GGTCCTTTTCACCAGCAAGCT	94
GAPDH	TGCACCACCAACTGCTTAGC	GGCATGGACTGTGGTCATGAG	87

## Supplementary Materials

The following are available online at https://www.mdpi.com/2072-6694/12/11/3138/s1, Figure S1: Correlation analysis, Figure S2: HPLC purification of radiolabeled angiotensin II, Figure S3: Quantification of autoradiography by an alternative method, Figure S4: In vitro receptor autoradiography of pancreatic and small-intestinal NET tissues, Figure S5: In vitro receptor autoradiography of pancreatic and small-intestinal control tissues, Figure S6: In vivo near-infra red fluorescent (NIRF) imaging with AGTR1-targeting valsartan–ITCC, Table S1: Screening library compound list, Table S2: Confirmed hits from screening, Table S3: Clinical characteristics of the tissues analyzed by receptor autoradiography.
